# Acute non-occlusive mesenteric ischemia of the small bowel in a patient started on hemodialysis: a case report

**DOI:** 10.1186/1757-1626-1-217

**Published:** 2008-10-06

**Authors:** Zachary Z Brener, Michael Bergman, Hyunsook K Ohm, James F Winchester

**Affiliations:** 1 Division of Nephrology, Beth Israel Medical Center, 1350 E. 17th Street, Baird Pav, 18th Floor, New York, NY, USA; 2 Department of Medicine, Golda Campus – Hasharon, Rabin Medical Center, 7 Keren Kayemet Street, Petah – Tikva, 49372, Israel; 3 Department of Pathology, New York Community Hospital, 2525 Kings Highway, Brooklyn, NY, 11229, USA

## Abstract

**Background:**

Non-occlusive mesenteric ischemia is not uncommon in chronic hemodialysis patients and is the major cause of an acute abdomen in this population. Intensive ultrafiltration and intradialytic hypotension are usually the precipitation factors. A definite diagnosis is usually late and associated with high mortality. We present a rare case of a patient who developed abdominal symptoms during his first week on HD without having obvious hypotensive episodes.

**Case presentation:**

A 76-year-old man was admitted with pulmonary edema and renal failure developed abdominal symptoms during his first week on hemodialysis without having obvious hypotensive episodes. Abdominal diagnostic procedures were all unrevealing. Mesenteric ischemia was diagnosed during laparoscopy done on the basis of physical findings and clinical suspicion. Ischemic small bowel of the distal ileum was resected and histopathology examination of the small bowel demonstrated transmural ischemic necrosis with hemorrhages and non-occluded mesenteric artery. Patient maid a steady recovery, and was discharged on the 11^th ^post-operative day.

**Conclusion:**

Mesenteric ischemia should be systematically suspected in dialysis patients experiencing even mild and nonspecific abdominal symptoms with or without hemodialysis-induced hypotensive episodes. Identification of patients at risk and prevention of intradialytic hypotension may help to reduce the incidence of this potentially fatal complication in hemodialysis patients.

## Background

Acute mesenteric ischemia (AMI) was first described by Antonio Beniviene in the 15^th ^century and later by Virchow in the 19th century [1]. AMI is a syndrome in which inadequate blood flow through the mesenteric circulation causes ischemia and eventual gangrene of the bowel wall. Arterial disease can be subdivided into non-occlusive mesenteric ischemia (NOMI) and occlusive mesenteric arterial ischemia (OMAI). NOMI was first recognized as a subtype of AMI in the 1950s. NOMI is a predominant feature in hemodialysis (HD) patients [2] and is usually associated with circulatory failure due to hypotension, acute heart failure and use of vasoconstrictors [2,3]. Recent publications indicated that the frequency of NOMI is increasing in HD population [2,4-6], with the frequency estimated to be as high as 1.9% per patient-year, comparing to 0.2% per patient-year in non-hemodialysis adults [7], since more elderly patients with atherosclerotic cardiovascular disease are being chronically dialyzed. The diagnosis of NOMI is difficult and requires a high level of suspicion since a delay in an early diagnosis results in a high mortality rate [2,4].

We present a rare case of a 76-year-old man who developed abdominal symptoms during his first week on HD without having obvious hypotensive episodes. Abdominal diagnostic procedures were all unrevealing. Laparotomy showed NOMI of small intestine.

## Case presentation

A 76-year-old man with known history of chronic kidney disease, cardiac disease, hypertension and chronic obstructive lung disease was admitted to our hospital with pulmonary edema. The patient had kidney disease of unknown etiology with baseline serum creatinine of 4.0 mg/dL (calculated glomerular filtration rate, 14 ml/min/1.73 m^2^) and was not on dialysis.

**Figure 1:**
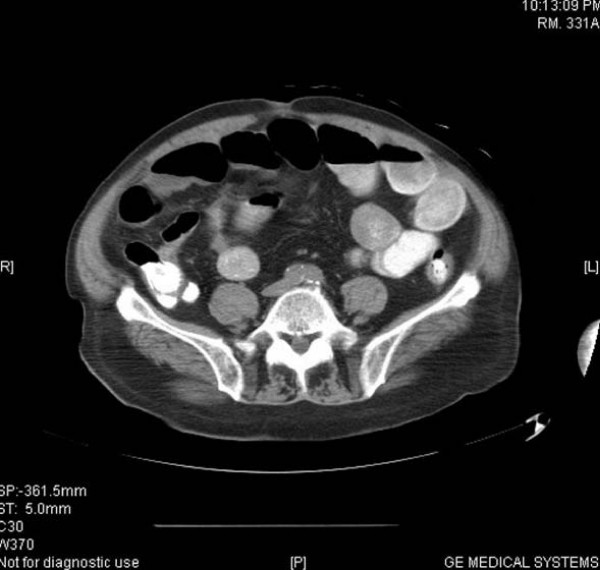
Abdominal CT scan with intravenous contrast showed mild ascites and increased small bowel distention.

**Figure 2:**
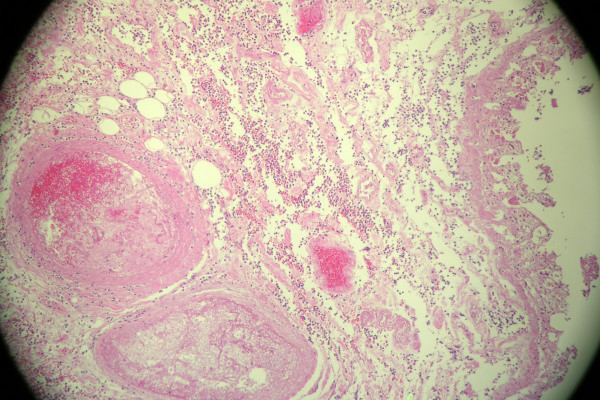
Histopathologic examination of the patient's small bowel showed transmural ischemic necrosis with hemorrhages and non-occluded mesenteric artery.

On examination, blood pressure was 170/75 mm Hg and respiratory rate 24/min and laboured. He required accessory muscles for respiration. There were bilateral rales over both lung fields. His heart and abdominal examination was not remarkable and there was no peripheral edema. Serum sodium was 143 mEq/L; potassium, 5.9 mEq/L; blood urea nitrogen, 70 mg/dL; serum creatinine, 5,0 mg/dL; albumin, 3.7 g/dL. Hemoglobin level was 11.8 g/dL, hematocrit 35%, and white blood cell (WBC) count was 12 × 10^3^/μL. Urinalysis showed proteinuria, with protein of 30 mg/dL, and moderate blood, with 20 to 30 red blood cells/high-power field. Chest X-ray showed pulmonary edema. Renal ultrasound revealed small right kidney. Patient refused dialysis treatment and was admitted to ICU. On the third hospital day he remained volume overloaded and hypertensive despite of intravenous furosemide, his creatinine rose to 5.5 mg/dL, urea nitrogen to 91 mg/dL, and patient consented to HD. After two HD sessions, his volume status and blood pressure improved but the patient complained of mild right upper quadrant abdominal pain and nausea at the end of his second treatment. He was afebrile and denied having diarrhea, chills or vomiting. On examination, his abdomen was slightly distended with hypoactive bowel sounds and right upper quadrant tenderness. An abdominal computer tomography (CT) scan revealed distended gallbladder with sludge and diverticulosis of the sigmoid colon. Upper endoscopy (EGD) showed gastritis. Intravenous antibiotics with ceftriaxone and metronidazole started for presumed acute acalculous cholecystitis. Over next week the patient had few hypotensive episodes during HD with systolic blood pressure of 70–80 mm Hg associated with marked increase of abdominal pain. Rebound abdominal tenderness and leukocytosis with WBC count of 24.5 × 10^3^/μL with 90% neutrophiles noted. Repeated abdominal CT scan with intravenous contrast showed mild ascites and increased small bowel distention (Figure 1). Hepatobiliary (HIDA) scan was unremarkable. A diagnostic laparoscopy converted to exploratory laparotomy revealed ischemic small bowel of the distal ileum with at least two areas of necrosis that was resected with anastomosis and insertion of feeding catheter per jejunostomy tube. Histopathology examination of the small bowel demonstrated transmural ischemic necrosis with hemorrhages and non-occluded mesenteric artery (Figure 2). Patient maid a steady recovery, and was discharged on the 11^th ^post-operative day.

## Discussion

In recent years, NOMI has become increasingly recognized as a highly dangerous complication in dialysis patients. Patients with end-stage renal disease have many risks factors for the development of mesenteric ischemia. These include generalized atherosclerosis, congestive heart failure, diabetes mellitus and increased time on dialysis [2], the use of digoxin, a drug with a potential vasoconstrictive effect on mesenteric vessels [3], and the need to remove large volumes of fluid from the intravascular space leading to relative hypovolemia and hypotension. Hypotension, especially repeated episodes, is the most important and immediate precipitating factor for NOMI patients on dialysis [4]. In the previous studies [2,5], all patients had severe hypotension before the onset of abdominal pain. In the case/control study by Bassilios et al. [4], the main difference between the 2 groups was a significant reduction in both systolic and diastolic blood pressure prior to the development of abdominal pain in the affected patients.

Mesenteric ischemia may appear more frequently among dialysis patients than in the non-dialysis population. In the general population, mesenteric ischemia is of an occlusive type, usually due to atherosclerotic thrombosis of the proximal portion of the superior mesenteric artery affecting the left colon and sigmoid [6]. In contrast, HD patients show a non-occlusive type of mesenteric ischemia and lesions develop more frequently in the cecum and right colon [8]. This type of ischemia is precipitated by a severe reduction in mesenteric perfusion with secondary arterial spasm from such causes as severe hypotension during hemodialysis and myocardial infarction. The left colon maintains a better collateral circulation due to Drummond artery and is considered more resistant to ischemia [9].

The diagnosis of mesenteric ischemia is difficult and requires a high level of suspicion since a delay in an early diagnosis leads to a high mortality rate [2,5]. John et al. [2] demonstrated that NOMI was the correct initial diagnosis in only 6 out of 29 patients. Although previous studies described a high incidence of abdominal guarding upon presentation [2,4,5], mild abdominal signs do not rule out this diagnosis [4]. Pain usually begins in the right iliac fossa with diffuse guarding developing later [4,8], and often accompanied with fever, leukocytosis and metabolic acidosis [2,4]. Our patient developed mild abdominal symptoms during his second and third HD sessions without having hypotensive episodes with guarding, fever and leukocytosis appearing a week later when obvious falls in both systolic and diastolic blood pressure were noted during HD. Intensive ultrafiltration and optimizing patient blood pressure even without having obvious hypotensive episodes during the first few weeks after initiation of dialysis may represent an important factor in triggering mesenteric hypoperfusion and ischemic insults.

Angiography findings can confirm diagnosis of arterial occlusion and indicate NOMI when 'defoliated tree' that refers to the absence of contrast dye in the smaller mesenteric vessels is present [10]. Angiography should be performed at an early stage in a patient with suspected mesenteric ischemia and, after diagnosis has been maid, can be used to introduce vasodilator drugs, such as papaverine [10]. In recent study water-soluble opaque enema abdominal CT scan findings of thickening fatty tissues around the involved colonic segment together with gas in the colonic wall in 6 out of 8 patients led to early surgery [4]. Five of these 6 patients survived.

The known difficulty in diagnosing intestinal ischemia and the role of early, definitive surgery was exemplified in the group of 12 chronic HD patients admitted to the John Hopkins Bayview Medical Center because of acute abdominal pain with leukocytosis [10]. Abdominal diagnostic procedures (CT scan, HIDA, ultrasound) were all unrevealing. No patient underwent preoperative angiography either because lack of clinical suspicion of mesenteric ischemia or because peritonitis became evident [7]. Like in case of our patient, all patients who were operated on had this done on the basis of physical findings and clinical suspicion. Only one patient with pancreatitis had the correct diagnosis made on presentation. The other 11 patients all had NOMI as the cause of their admission [7]. Mortality was high (45%) when surgery was delayed to after the first 24 h versus no death when this interval was reduced to below this critical period. Authors also concluded that in view of large number of patients reviewed (567) mesenteric ischemia could be a major cause of acute abdominal problems in HD patients.

## Conclusion

Mesenteric ischemia is a potentially fatal complication in chronic HD patients and is the major cause of an acute abdomen in this population. An early diagnosis is difficult to establish. A definite diagnosis is usually late and associated with high mortality. To our knowledge, this is a first report of NOMI that occurred at the fist week of chronic HD therapy and without hypotensive episodes. As our report illustrates, mesenteric ischemia should be systematically suspected in patients experiencing even mild abdominal symptoms during or after HD sessions, especially in elderly patients with atherosclerotic cardiovascular disease even without HD-induced hypotensive episodes. Identification of patients at risk and prevention of intradialytic hypotension may help to reduce the incidence of NOMI in chronic HD patients.

## Consent

Written informed consent was obtained from the patient for publication of this case report and accompanying images. A copy of the written consent is available for review by the Editor-in-Chief of this journal.

## Competing interests

The authors declare that they have no competing interests.

## Authors' contributions

ZZB and MB analyzed and interpreted the patient data. HKO performed histopathological studies. JFW was responsible for manuscript editing. All authors read and approved the final manuscript.
